# Does it come from tobacco? Young adults’ interpretations of the term “tobacco-free nicotine” in a cross-sectional national survey sample

**DOI:** 10.1371/journal.pone.0268464

**Published:** 2022-05-13

**Authors:** Meghan E. Morean, Krysten W. Bold, Danielle R. Davis, Grace Kong, Suchitra Krishnan-Sarin, Deepa R. Camenga

**Affiliations:** Department of Psychiatry, Yale University School of Medicine, New Haven, CT, United States of America; Medical University of South Carolina, UNITED STATES

## Abstract

**Background:**

“Tobacco-free” nicotine (TFN) e-cigarettes and nicotine pouches containing synthetic nicotine are increasingly available. The term TFN may lead to reduced risk perceptions and increased use intentions relative to tobacco-derived nicotine products. Effectively communicating messages about TFN may depend on the public’s ability to differentiate TFN from tobacco-derived nicotine. Our goals were to examine knowledge about the source(s) of nicotine in commonly used products and beliefs about what TFN means.

**Methods:**

In 2021 we surveyed 2464 young adults (18–25 years) online. Participants reported whether cigarettes, smokeless tobacco, e-cigarettes, and nicotine pouches contain nicotine that comes from tobacco (always, sometimes, never). Correct responses were “always” for cigarettes/smokeless and “sometimes” for e-cigarettes/pouches. Participants also reported “what [they] think TFN e-cigarettes/vapes contain” (nicotine only; tobacco only; both nicotine and tobacco; neither nicotine nor tobacco). We ran unadjusted and adjusted models examining correct responses for nicotine source and TFN contents by past-month product use status (cigarettes, smokeless, e-cigarettes, pouches).

**Results:**

Rates of correctly identifying nicotine source were modest (23.6% pouches—61.9% cigarettes). Except smokeless tobacco, using a given product was associated with identifying its nicotine source correctly in unadjusted models. Participants reported “TFN” means a product contains nicotine only (57.8%), tobacco only (10.8%), both (14.1%), or neither (17.1%).

**Conclusions:**

There is confusion about the source of nicotine in products, and many young adults incorrectly interpreted TFN to mean something other than containing nicotine but no tobacco. Regulatory efforts may be needed to restrict using the term “tobacco-free nicotine” on product labeling and advertising.

## Introduction

In 2016, the US Food and Drug Administration (FDA) deemed electronic cigarettes to fall under its tobacco regulatory authority because they contained tobacco-derived nicotine [1, 2]. In recent years, e-cigarette manufacturers and nicotine pouch manufacturers began marketing “tobacco-free nicotine” (TFN) products which they claim contain synthetic nicotine that is not tobacco-derived [2]. Until March 2022, there were considerable concerns that TFN did not legally fall under the FDA’s definition of a tobacco product [1] and that manufacturers were using TFN to circumvent compliance with FDA regulations. For example, in 2020 the FDA prioritized enforcement actions against flavors other than menthol or tobacco for use in cartridge/pod-based e-cigarettes [3]. However, TFN e-cigarettes often come in flavors like fruit that otherwise would be prioritized for enforcement. Now that the FDA has the authority to regulate synthetic nicotine as a tobacco product, restrictions on flavors for use in certain devices (i.e., pod/cartridge devices) may be implemented to align with how tobacco-derived nicotine (TN) e-cigarettes currently are regulated. However, other issues with TFN may remain. Importantly, the FDA has not released any information about if or how use of the term “tobacco-free nicotine” itself in product marketing may be regulated. Although public health research on TFN e-cigarettes is extremely limited and little is known about the health effects of synthetic nicotine relative to tobacco-derived nicotine (e.g., addition potential) [4], findings suggest that the label “Tobacco-Free Nicotine” decreases e-cigarette risk perceptions and increases use intentions among young adults [5]—a population with high rates of e-cigarette use and susceptibility [5]. If synthetic nicotine is found to have comparable or elevated risks relative to tobacco-derived nicotine, education efforts that correct misperceptions of the term TFN may become warranted. Further, if use of the term TFN is confirmed to be misleading (e.g., elicits reduced risk perceptions that are unfounded, leads consumers to believe that a product contains no nicotine), this would support regulatory efforts that limit or prohibit the use of the term.

Before messaging can be developed or regulatory action taken, it is critical to understand the public’s ability to differentiate TFN from tobacco-derived nicotine products and what they understand TFN products to contain. In the current study, we examined young adults’ baseline level of knowledge about the source(s) of nicotine in commonly used nicotine/tobacco products including cigarettes, smokeless tobacco, e-cigarettes, and nicotine pouches. We then directly evaluated their understanding of the term “tobacco-free nicotine” as it applies to e-cigarettes.

We expected that most participants would report that cigarettes and smokeless tobacco contain nicotine that comes from tobacco. Given the novelty of TFN products, we did not make any hypotheses about the extent to which participants would know that e-cigarettes and nicotine pouches may contain either tobacco-derived or synthetic nicotine. Finally, we expected that the majority of participants would correctly interpret the term TFN to mean that a product contains only nicotine (with no tobacco). However, we expected to observe some confusion around the term (e.g., believing TFN contains no nicotine or tobacco) based on the juxtaposition of the terms “tobacco, “free,” and “nicotine.”

## Materials and methods

### Participants and procedure

All study procedures were approved by the Yale University Institutional Review Board. From September-October 2021, 2464 US young adults ages 18–25 years old participated in an anonymous online survey (eligibility rate = 18.9%; 2,464/13,014). Panelists were recruited and compensated directly by Qualtrics^TM^ Online Sample (QOS), a secure market research service operated by *Qualtrics*, *Inc*^TM^. QOS sent targeted emails to participants who were likely to be eligible based on their responses to previous QOS surveys (e.g., smoking status). Interested participants clicked on a link that directed them to the eligibility questions (see Measures section), and, if eligible, they were directed to the informed consent form and central study questions. Consent was indicated by marking a box (no/yes) indicating one’s desire to participate to preserve the anonymous nature of the survey. Basic eligibility for this study included reporting on sex, ethnicity/race, and lifetime and past-30-day use of a variety of nicotine/tobacco products (see Measures section). Current tobacco/nicotine product users were oversampled. Quotas were set to ensure diversity in terms of sex (approximately 50/50), ethnicity/race (no more than 40% non-Hispanic white), and across four past-30-day tobacco/nicotine product use groups (goal of 25% per subsample; groups listed below). However, recruiting current exclusive users of tobacco products other than e-cigarettes proved difficult, so the final sample reflected no current tobacco product use (29% [14.3% never users; 14.7% lifetime users), current exclusive e-cigarette use (26.1%), current use of non-e-cigarette tobacco products (15.6%), and current dual-use of e-cigarettes and any other tobacco product(s) (29.3%).

### Measures

#### Screening questions/demographics

Participants reported on age, biological sex (female/male), Hispanic ethnicity (no/yes), and Race (select all that apply from White, Black/African American, Asian, Native American, Pacific Islander or Native Hawaiian, Other). Participants then reported on lifetime use of the following (no/yes): disposable pod vape, e-hookah, cig-a-like, vape pen, JUUL, a rechargeable pod device other than JUUL, Mod/APV (Advanced Personal Vaporizer), hookah, cigar/cigarillo, smokeless tobacco, and nicotine pouch. Each product was accompanied by a description and image showing examples of products in the category. Participants who endorsed lifetime use of a product reported on current use (i.e., number of days out of the past 30 days). Responses to these questions were used to categorize participants into one of the four past-30-day tobacco product use groups. Participants also reported on subjective financial situation as a proxy for socioeconomic status (responses: “do not meet basic expenses,” “just meet basic expenses,” “meet needs with a little left,” “live comfortably”) [6].

#### Knowledge of the nicotine source of common nicotine/tobacco products

Participants reported how often tobacco cigarettes, smokeless tobacco, e-cigarettes, and nicotine pouches, respectively, “contain nicotine that comes from the tobacco plant” (always, sometimes, never). Correct responses were “always” for cigarettes/smokeless and “sometimes” for e-cigarettes/pouches.

#### Knowledge of the contents of tobacco-free nicotine e-cigarettes

Participants viewed the following: “Some e-cigarettes are described as containing ‘tobacco-free nicotine.’ What do you think tobacco-free nicotine vapes/e-cigarettes contain?” (nicotine only [no tobacco]; tobacco only [no nicotine]; nicotine and tobacco; neither nicotine nor tobacco). The correct response was “nicotine only [no tobacco].”

### Analytic plan

We ran descriptive statistics and chi-square analyses by product use status (e.g., past-month e-cigarette use [no/yes]) in relation to correct responses about product nicotine source and TFN contents. We subsequently ran five adjusted binary logistic regression models with correctly identifying the nicotine source for each product and the contents of TFN e-cigarettes as the dependent variables. Independent variables included past-month use of cigarettes, e-cigarettes, smokeless tobacco, and nicotine pouches, respectively (all no/yes). Age, sex, Hispanic ethnicity (no/yes), white race (no/yes), black race (no/yes), and subjective financial status were included as covariates. To account for running multiple models, the threshold for statistical significance for each model was set at *p* < .01. There were no missing data.

## Results

Please see Table 1 for a summary of all study variables. Participants had an average age of 21.17 (SD = 2.30) years, and there was variability by sex, ethnic/racial background, and subjective financial status. Rates of lifetime product use ranged from 11.1% for nicotine pouches to 73.5% for e-cigarettes. Rates of past-month nicotine/tobacco product use were 55.4% e-cigarettes, 25.5% cigarettes, 20.4% cigar/cigarillo, 16.3% hookah, 6.4% smokeless tobacco, and 6.4% nicotine pouch.

**Table 1:** Participant demographics and central study variables.

	M (SD) or Percent
Age	21.17 (2.30)
Sex (Male)	48.0
Hispanic	34.0
White	30.5
Black	22.1
Financial Status	
Do not meet basic expenses	17.5
Just meet basic expenses	36.9
Meet needs with a little left over	23.1
Live comfortably	22.6
Lifetime Product Use	
Cigarettes	40.1
E-cigarettes	73.5
Smokeless Tobacco	12.4
Nicotine Pouch	11.1
Past-Month Product Use	
Cigarettes	25.5
E-cigarettes	55.4
Smokeless Tobacco	6.4
Nicotine Pouch	6.4
Nicotine in Cigarettes	
Never from Tobacco	24.3
Sometimes from Tobacco	13.8
Always from Tobacco*	61.9
Nicotine in E-cigarettes	
Never from Tobacco	25.2
Sometimes from Tobacco*	46.6
Always from Tobacco	28.2
Nicotine in Smokeless Tobacco	
Never from Tobacco	31.6
Sometimes from Tobacco	16.3
Always from Tobacco*	52.1
Nicotine in Nicotine Pouches	
Never from Tobacco	35.4
Sometimes from Tobacco*	23.6
Always from Tobacco	41.0
Contents of TFN E-cigarettes	
Nicotine only (no tobacco)*	57.8
Tobacco only (no nicotine)	10.8
Nicotine and tobacco	14.3
No nicotine, no tobacco	17.1

*Note*.

*indicates the correct response.

Rates of correctly identifying nicotine source were modest (range: 23.6% [pouches] to 61.9% [cigarettes]), and there was variability in terms of incorrect responses for each product (Table 1). For example, for e-cigarettes, 25.2% of participants thought e-cigarettes contain nicotine that never comes from tobacco and 28.2% thought e-cigarettes contain nicotine that always comes from tobacco.

**Fig 1:**
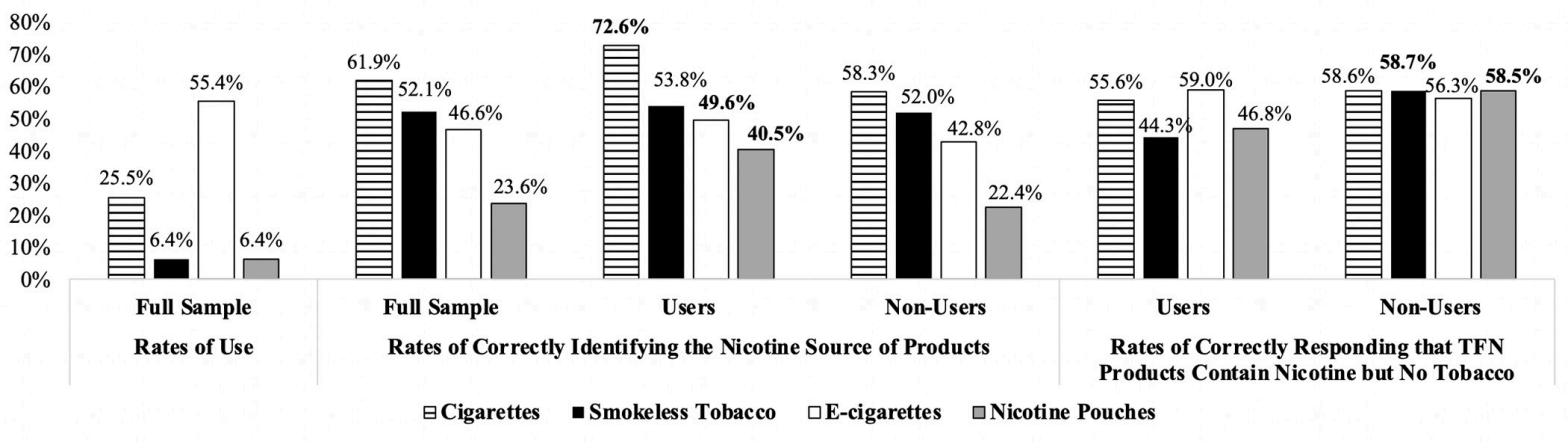
Rates of product use, correctly identifying nicotine source, and correct identifying the content of tobacco-free nicotine products. Bolded font indicates a significant difference in response between product users and non-users at *p* < .001. TFN = tobacco-free nicotine. The overall rate of correctly responding that tobacco-free nicotine products contain nicotine but not tobacco was 57.8%. The bars to the far right show the percentages of correctly responding that tobacco-free nicotine products contain nicotine but not tobacco by product use status.

Except for smokeless tobacco, use of a given substance was significantly associated with identifying its nicotine source correctly in unadjusted models (Fig 1). For example, 72.6% of cigarette smokers correctly responded that the nicotine in cigarettes always comes from tobacco compared to 58.3% of non-smokers. In the adjusted models (Table 2), there were no significant effects of past-month use of a given product on correctly identifying its nicotine source. With regard to demographics, white participants were more likely to correctly indicate that cigarettes and smokeless tobacco always contain nicotine from tobacco. In contrast, Black individuals were less likely to correctly indicate that smokeless tobacco contains nicotine from tobacco.

**Table 2:** Predictors of correctly identifying the nicotine source of cigarettes, e-cigarettes, smokless tobacco, and nicotine pouches and correctly identifying the content of tobacco-free nicotine e-cigarettes.

	Cigarettes							E-cigarettes						
	B	S.E.	Wald	Sig.	OR_adj_	95% CI		B	S.E.	Wald	Sig.	OR_adj_	95% CI	
Age	0.00	0.03	0.01	0.940	1.00	0.94	1.06	0.00	0.03	0.02	0.883	1.00	0.95	1.05
Male Sex	-0.03	0.13	0.06	0.802	0.97	0.75	1.25	-0.07	0.12	0.31	0.577	0.94	0.75	1.18
Hispanic	-0.23	0.14	2.74	0.098	0.79	0.60	1.04	-0.22	0.12	3.16	0.076	0.80	0.63	1.02
White	0.44	0.17	7.01	0.008	1.55**	1.12	2.14	0.16	0.15	1.13	0.288	1.17	0.88	1.56
Black	-0.38	0.18	4.45	0.035	0.68	0.48	0.97	-0.07	0.17	0.15	0.696	0.94	0.68	1.30
Financial Status	0.02	0.07	0.07	0.797	1.02	0.89	1.16	-0.02	0.06	0.13	0.718	0.98	0.87	1.10
Past Month Product Use														
E-cigarette	-0.37	0.14	7.27	0.007	0.69**	0.53	0.91	0.10	0.12	0.80	0.373	1.11	0.88	1.40
Cigarette	0.24	0.16	2.19	0.139	1.26	0.93	1.73	0.14	0.14	1.13	0.288	1.15	0.89	1.51
Nicotine Pouch	-0.45	0.33	1.85	0.174	0.64	0.33	1.22	-0.58	0.31	3.57	0.059	0.56	0.30	1.02
Smokeless Tobacco	0.08	0.36	0.05	0.820	1.09	0.53	2.22	0.40	0.33	1.51	0.220	1.49	0.79	2.82
Constant	1.15	0.67	2.96	0.085	3.16			0.19	0.58	0.10	0.749	1.21		
	Model. Chi-square (10) = 43.78, Nagelkerke R2 = 0.05, *p* < 0.001							Model. Chi-square (10) = 11.83, Nagelkerke R2 = 0.01, *p =* 0.297						
	Smokeless Tobacco							Nicotine Pouches						
	B	S.E.	Wald	Sig.	OR_adj_	95% CI		B	S.E.	Wald	Sig.	OR_adj_	95% CI	
Age	0.00	0.03	0.01	0.933	1.00	0.95	1.05	-0.04	0.03	1.67	0.197	0.96	0.91	1.02
Male Sex	0.01	0.12	0.00	0.955	1.01	0.79	1.28	0.32	0.13	5.90	0.015	1.37	1.06	1.77
Hispanic	-0.25	0.13	3.70	0.055	0.78	0.61	1.01	0.07	0.14	0.24	0.628	1.07	0.82	1.40
White	0.46	0.15	8.98	0.003	1.58**	1.17	2.13	0.18	0.17	1.15	0.283	1.20	0.86	1.65
Black	-0.49	0.17	8.38	0.004	0.61**	0.44	0.85	-0.32	0.19	2.75	0.097	0.73	0.50	1.06
Financial Status	0.03	0.06	0.30	0.582	1.03	0.92	1.17	0.07	0.07	1.28	0.259	1.08	0.95	1.22
Past Month Product Use														
E-cigarette	0.12	0.12	0.92	0.337	1.13	0.88	1.43	0.02	0.13	0.03	0.866	1.02	0.79	1.33
Cigarette	-0.16	0.14	1.29	0.257	0.85	0.65	1.12	0.13	0.15	0.78	0.378	1.14	0.85	1.53
Nicotine Pouch	-0.45	0.31	2.03	0.154	0.64	0.35	1.18	-0.11	0.33	0.12	0.730	0.89	0.47	1.71
Smokeless Tobacco	0.10	0.33	0.08	0.773	1.10	0.57	2.12	0.60	0.33	3.21	0.073	1.82	0.95	3.48
Constant	0.48	0.61	0.60	0.438	1.61			-0.66	0.66	1.00	0.316	0.52		
	Model. Chi-square (10) = 48.23, Nagelkerke R2 = 0.05, *p <* 0.001							Model. Chi-square (10) = 24.88, Nagelkerke R2 = 0.03, *p =* 0.006						
	Tobacco-Free Nicotine E-cigarettes													
	B	S.E.	Wald	Sig.	OR_adj_	95% CI								
Age	-0.07	0.03	5.67	0.017	0.94	0.88	0.99							
Male Sex	-0.06	0.13	0.24	0.628	0.94	0.73	1.21							
Hispanic	-0.52	0.14	14.40	0.000	0.59***	0.45	0.78							
White	0.53	0.16	10.89	0.001	1.70**	1.24	2.33							
Black	-0.35	0.18	3.95	0.047	0.70	0.50	1.00							
Financial Status	0.03	0.07	0.22	0.639	1.03	0.91	1.17							
Past Month Product Use														
E-cigarette	0.30	0.13	5.23	0.022	1.35	1.04	1.75							
Cigarette	-0.33	0.15	4.95	0.026	0.72	0.54	0.96							
Nicotine Pouch	-0.05	0.34	0.02	0.885	0.95	0.49	1.85							
Smokeless Tobacco	-0.74	0.34	4.85	0.028	0.48	0.25	0.92							
Constant	2.25	0.66	11.69	0.001	9.50									

Model. Chi-square (10) = 65.44, Nagelkerke R2 = 0.07, *p <* 0.001

While a majority of participants accurately interpreted “tobacco-free nicotine” to mean containing nicotine but no tobacco (57.8%), others misinterpreted TFN products as containing tobacco only (10.8%), both nicotine and tobacco (14.1%), or neither nicotine nor tobacco (17.1%). Product users were not more likely than non-users to correctly report that TFN contains nicotine without tobacco in unadjusted (Fig 1) or adjusted models (Table 2). With regard to demographics, white participants were more likely and Hispanic individuals were less likely to correctly indicate that TFN e-cigarettes contain nicotine but no tobacco.

## Discussion

This is the first study of which we are aware to assess understanding of the nicotine sources of common nicotine/tobacco products and to evaluate directly what young adults interpret the term “tobacco-free nicotine” to mean. While people can interpret the term TFN solely based on the words that comprise it: “tobacco,” “free,” and “nicotine,” the ability to understand the term may be linked to a broader understanding of the source of nicotine in tobacco products. For instance, one may only be able to truly understand the meaning and potential implications of TFN if they understand its comparator, tobacco-derived nicotine (or nicotine that comes from tobacco). This would involve having a basic understanding of what types of nicotine common tobacco products contain (e.g., tobacco cigarettes contain nicotine from tobacco).

Consistent with our hypotheses, the majority of participants knew that cigarettes (61.9%) and smokeless tobacco (52.1%) always contain nicotine from tobacco. For both e-cigarettes and nicotine pouches, a minority of participants correctly identified the nicotine source (e-cigarettes [49.6%]; pouches [40.5%]). Although rates of correct responses were higher among current product users in unadjusted models, past-month product use was not associated with correct responses in the adjusted models. Where demographic differences were observed, white participants were more likely to correctly identify the nicotine source for cigarettes and smokeless tobacco than were their non-white counterparts, while black individuals were less likely to correctly indicate that smokeless tobacco contains nicotine from tobacco. Given the novelty of the current research question, future research is needed to understand racial differences in understanding the nicotine source of common products. While additional research is needed, the overall pattern of findings suggests that young adults’ ability to differentiate the source of nicotine in common products (i.e., tobacco-derived versus synthetic) is lacking. Although this was not tested directly in the current study, given the low rates of correctly identifying the nicotine source of e-cigarettes and nicotine pouches (the two products that regularly are available with TFN), the findings also suggest that product users and non-users likely are not relying on their knowledge of the source of nicotine in tobacco products to interpret the term TFN.

Consistent with our hypothesis, the majority of participants (57.8%) correctly interpreted the term TFN to mean an e-cigarette containing nicotine but no tobacco. However, 42.2% incorrectly interpreted the term. Of greatest concern, 17.1% of young adults in our sample interpreted TFN to mean that a product does not contain nicotine or tobacco. Of note, engaging in past-month product use was not associated with correctly identifying the contents of TFN e-cigarettes in unadjusted or adjusted models. In fact, where differences emerged, individuals who had not used a product in the past month (i.e., smokeless tobacco and nicotine pouches) were more likely to answer correctly. This finding was unexpected for nicotine pouch users, a product that regularly contains TFN, but may be expected for users of a product like traditional smokeless tobacco that never contains synthetic nicotine. These findings also may be driven by the fact that nicotine pouches and smokeless tobacco were the least commonly used products and that product use was not mutually exclusive (e.g., a non-user of smokeless tobacco may have used another product like e-cigarettes). Thus, correct responding among non-users may have been driven by participants having greater familiarity and experience with other products like e-cigarettes that are available in TFN. Where ethnic/racial effects emerged, white participants were more likely and Hispanic participants were less likely to correctly indicate that TFN e-cigarettes contain nicotine but no tobacco. Again, given the nascency of research on TFN, additional research is needed to understand ethnic/racial differences in interpretation of the term TFN. While further work is needed, if study findings are replicated this would suggest that, although non-combustible nicotine products like e-cigarettes may be less harmful alternatives to combustible products like cigarettes [7] irrespective of the type of nicotine they contain, manufacturers’ use of the term TFN may be misleading to the public. As such, regulatory efforts may be needed to prevent inclusion of the term on product packaging or in advertising.

The study findings should be considered alongside its limitations. First, participants were young adult Qualtrics panelists, which may limit generalizability, although the sample was diverse with regard to sex, ethnicity/race, and financial status. Second, current users of nicotine/tobacco products were over-represented in our sample, so the rates presented in the current paper cannot be interpreted as reflecting prevalence. That said, the results suggest that confusion exists even among current nicotine/tobacco product users. Third, this study was designed to provide a broad overview of young adults’ understanding of nicotine source and interpretation of TFN. However, prior familiarity with each product was not assessed which could have impacted the study findings. For example, if a participant heard about nicotine pouches for the first time in the study, they may have incorrectly identified the nicotine source due to lack of familiarity with the product or confusion between nicotine pouches and smokeless products like snus (although snus was included in the product description for smokeless tobacco). Related, the nicotine product landscape, in general, is rapidly expanding, and products that are designed to mimic others may lead to confusion for consumers. For example, Black Buffalo pouches are designed to mimic smokeless tobacco but do not actually contain tobacco leaves (instead they contain “edible green leaves” [https://blackbuffalo.com]). Because Black Buffalo contains tobacco-derived nicotine, confusion as to whether Black Buffalo is a smokeless tobacco product would not impact the correct response in this case (i.e., always contains nicotine from tobacco). However, products like Black Buffalo may make responding to questions like those posed in the current study difficult especially for those who are unfamiliar with the specific products. Fourth, the question assessing TFN contents was limited to e-cigarettes, and future research should assess participants’ understanding of the contents of other TFN products, such as nicotine pouches. Further research also is needed to examine additional participant and/or product characteristics that may influence understanding of nicotine sources and the term TFN. Fifth, the response options for the question assessing knowledge of TFN contents could have been confusing to some participants. For example, the correct response was that TFN e-cigarettes contain “nicotine only (no tobacco).” If participants interpreted this to mean solely containing nicotine without tobacco, other flavors, or other constituents, this may have introduced some confusion. That said, this concern should be mitigated by the fact that any confusion would apply equally to each response option (e.g., tobacco only [no nicotine]). Sixth, although the goal of the current study was to obtain a baseline understanding of the term TFN as it applies to e-cigarettes, consumers may encounter additional information about a product in the real world. For example, TFN e-cigarette packaging contains the FDA-mandated nicotine warning (“This product contains nicotine. Nicotine is an addictive chemical”). Some of the misunderstanding observed in the current study (i.e., 17.1% of participants thought that TFN e-cigarettes contained neither nicotine nor tobacco) may be mitigated by viewing the nicotine warning on product packaging. Thus, future research that exposes participants to real product packaging is needed to determine the extent to which confusion is reduced when participants have access to all information available on product packaging. Finally, the current study focused on the basic interpretation of the term “tobacco-free nicotine.” It is not clear how correct versus incorrect interpretations of the term impact risk perceptions or product use, including the use of TFN products, and this would have implications for what next steps are needed in terms of education and/or regulatory efforts. For example, if lowered risk perceptions are found to be driven by individuals who incorrectly interpret the term “tobacco-free nicotine,” this would suggest that corrective educational efforts may help to remedy the problem. However, if lowered risk perceptions are observed even among those who correctly interpret the term “tobacco-free nicotine,” this would suggest that the term itself should be prohibited because it is deceptive.

In sum, the current study indicates that even nicotine/tobacco product users are confused about the source of nicotine in common products. Further, 42.2% of young adults in our sample incorrectly interpreted TFN to mean something other than containing nicotine but no tobacco. Given that TFN products are increasing in popularity, it will be important for education and/or regulatory efforts to consider the impact of public misunderstanding of the source of nicotine and “tobacco-free nicotine” labeling.

## Supporting information

S1 Data (XLSX)
